# Mr. Pulley on the Case of Ann Foulkes

**Published:** 1813-12

**Authors:** John Pulley

**Affiliations:** Bedford


*To the Editor of the Medical and Physical Journal.*

SIR,

I CONGRATULATE you, the medical profession at large, and all lovers of truth, on the appearance of the case of Ann Foulkes, from the pen of Mr. Gibbon. It is now before the world, and all those whose mental age will admit of clear discernment, will not fail to observe, that a grosser act of imposition was never practised on the weakness and credulity of mankind. I would willingly proceed instantly to the consideration of it, but I am compelled previously to say a word or two in justification of myself. From the letter of Dr. Yeats, contained in the 175th Number of your

Journal, it seems most anxiously desired that my interference on the subject of Ischuria should be considered intrusive, as the matter originally commenced between the doctor and Mr. Gibbon. Now, when the difference of opinion arose between those gentlemen, the latter formally and professionally invited me to see the case, to inquire into its history, and to give my opinion upon it. I called, I saw, I investigated, and believed that deception was the order of the day. Subsequently and repeatedly I stated my opinion to Dr. Yeats, and he repeatedly pledged himself to prove the authenticity of the case. The case, *and nothing but the case*, was then pledged to be established; and to which, *and which alone*, the "written document,"* alluded to by the doctor in your Number for July, referred. By the way, I have now only to add, that the opinion which I shall hereafter give of urinous vomiting in general, was well known to Dr. Yeats before he published a single syllable on the subject.

I first saw Ann Foulkes, to examine her touching the reputed disease, a short time after her admission that her urine was again evacuated by its natural channel. She was then unwell, and confined chiefly to her bed. Her general look was such as I had known her to possess during several years past. She is thin and spare in body, constitutionally; and this natural scantiness of flesh is attached to her near relations, as well as to herself. At the above period, she was not less fleshy than I had known her to be several years before, when not complaining of indisposition; and though very thin, she had not the appearances of emaciation, either about her face, or any other part of her body. There was no flabby feel about her skin and muscles, and her breasts had more firmness and rotundity than are usually observed in women of her form. It is perfectly unnecessary for me to go through the whole case so amply detailed by Mr. Gibbon. I will remark only on a few points; but other circumstances cannot fail to strike the mind of every one, as tending to throw discredit on it altogether. Her appetite must have been moderately good, and her digestion tolerable, or her frame could not have been duly nourished, and her fæces healthy and natural. Great emaciation is the consequence of severe and dangerous diseases, especially with those of long continuance; but Ann Foulkes, after having weathered the storm six months, was as fleshy probably as

* The document was signed by Dr. Kerr, of Northampton; Dr. Alvey, of St. Neots; Mr. Short and Mr. Gibbon, of Bedford; and myself.

at at any period of her life. Is it not wonderfully singular that emetics should have the power of almost invariably coaxing the urine to and from the stomach?—I think seriously, and feel the most indignant desire to reprobate the imposition; and yet I cannot altogether bring my mind to write gravely on the subject. Half a pint of urine, clear and unsullied, without the slightest possible admixture of any foreign matter, was shown to Mr. Gibbon by the hypocritical mother of the patient, as coming from the stomach *under the operation of an emetic!* But, away with the emetic, and all thought about it. Even were it admitted as a fact incontrovertibly established, that *pure* urine could be ejected by vomiting, I deny that it could be produced entirely free from other matter. Some particles of gastric juice and mucus must have escaped with it; and if not these, some particles of saliva. The action of vomiting INVARIABLY increases the action of the vessels of the salivary glands; and it must come home to the recollection of all your readers, that they never retched without evacuating some quantity of the salivary fluid. It is remarkable that, throughout this case of six long months duration, the catheter was never proposed for trial, and never introduced; although at no period did a symptom exist to render its use improper.* It is also worthy of remark that, *in the pure spirit of piety and truth*, two women declared on oath before a magistrate that they saw urine actually vomited from the stomach; though these women had previously asserted before two medical gentlemen of this town, that they never witnessed any thing of the kind. I cannot suppose that any man, after a serious perusal of the case at large, will regard it as a fact of urinous vomiting. I enter my protest against it, and shall bear the penalty of want of credence, whatever it may be, with fortitude, but not with submission. I have read of twenty thousand devils dancing a saraband on the point of a needle; and when I shall discern those infernal gentry tripping on the light fantastic toe and mingling in the dance, then, and not till then, shall I believe in the urinary aberrations of Ann Foulkes.

Now, when urine is secreted, and any cause exists to obstruct its natural evacuation, absorption, in some degree, must be the inevitable consequence; and the fluid thus taken up must unavoidably be passed on by the absorbent channels to the blood-vessels. Having once entered the veins, it

* It must not be forgotten, that Mr. Gibbon strongly and repeatedly urged the introduction of the catheter, which was objected to on other grounds besides the dislike of the patient.

commixes with the blood, is diffused throughout it, and becomes subject to the laws of the circulation. I admit, therefore, that *urinous particles may* and *must* have their exit from *any* and from *every* exhalent surface: indeed, under Ischuria, life could not be maintained without it; and I feel so strongly impressed on the subject, that, whenever a fatal case of lengthened duration shall occur, I will venture to affirm that, on dissection, *any* collection of a fluid, in *any* part of the body, would possess both the smell and taste of urine. But, when urine is once absorbed, I deny that, *as urine*, it can be evacuated. It must pass from every pore with the natural, or rather corrupted, fluid of the parts, in an altered and adulterated condition. From its quantity and unusual retention, it must be expected to preserve both its taste and smell; but as *pure urine* it cannot be discharged. And, with my view of the subject, it is utterly impossible, under the phenomena of Ischuria, that the stomach, or any other part, should be found to have the urine regularly, uniformly, and systematically, directed *to it*, and *it alone*, for weeks and months together.

But what avails all this.—We are told that vomiting of urine can take place for weeks, months, nay even years, together; and *facts*, "stubborn" and "indisputable," are marshalled and arranged against us, of modern date, and drawn from the records of earlier time. FACTS are indeed stubborn things! and I am well aware that it is an ungracious task to question the veracity of statements, when introduced with specious front and bold assertion, and more especially when extracted from the *dead*, whose remains we are taught to touch with tenderness and awe. I desire not wantonly to doubt the word of any man; but surely, when I reflect on the writings of some medical authors, I am justly entitled to withhold my credence on matters which appear to me more supernatural than reasonable. In fact, were I called upon at this moment to credit every thing which has been published on medical subjects, I should be compelled almost to believe that a sovereign remedy existed for the cure of every disease. The press has often groaned under its labors to announce the birth of weighty remedies for formidable complaints; but when such helps have been submitted to the test of fair and honest experiment, they have usually been condemned for their imbecility and inertness. Whenever *very extraordinary* occurrences arise in pathology, the first duty of a medical man is, to doubt; his second duty, thoroughly to investigate; nothing ought to be granted; nothing ought to be published as true, *unless incontrovertibly witnessed and substantiated*. But it is a lamentable fact, that some some medical characters possess a thirst for novelty which never can be allayed, and an appetite for the marvellous of insatiable voracity; they catch reports and assertions as they fly, and, instead of calmly interposing in the flight, in aid of reason and of truth, are eager only to add a feather to the wing.

Since the case of Dr. Senter, taken from the Transactions of the College of Physicians at Philadelphia, seems to be triumphantly brought forward as the most positive and decisive to establish the truth of urinous vomiting, I beg leave to go a little into the consideration of it. It appears that Lucy Foster, the subject of it, labored under ischuria more than three years; that, during the continuance of her disease, her urine was constantly secreted by the kidnies, and regularly passed to the bladder; but that she was totally incapable of evacuating it by its natural passage, except in two instances when under the influence of fright. She never failed to vomit urine in thirty or thirty-six hours, unless drawn off by the catheter; and to put this beyond a doubt, was often watched at such times as the recurrence of the vomiting was expected. On such occasions, the bladder was discovered to be full, hard, and tender to the touch; and the urine vomited, when compared with the urine drawn off, was found in every respect exactly similar. During the last twenty months of her complaint, she sometimes vomited gravelly matter; such matter also was evacuated with the urine on the use of the catheter—and on comparing several drachms of the gravel from both sources, it was found the same in color and consistence. Other medical men attended this extraordinary case, and among them, Dr. Mason witnessed the vomiting both of urine and gravel. At one period, no urine was vomited for three days, nor did any pass on the introduction of the catheter; it then was expelled at the umbilicus: and when the disease had continued a length of time, urine passed often by the rectum, latterly sometimes with gravel. At one time the patient was so exhausted by her sufferings as not to be expected to outlive the month, then vomiting more sandy matter than at any other period; and throughout her disease, she labored under continual fever, had great loss of strength, and gradually became much emaciated. Death, at last, put an end to the complicated machinery of her urinary viscera!

I disbelieve entirely the reality of this case. On dissection, no cavity of the body contained a drop of urinous matter, not even the stomach and intestines; in fact, the bare smell of it is not mentioned. Several hydatids, the size of a wallnut, were attached to the Tubæ Fallopianæ, and the ovaria ovaria were enlarged to the magnitude of a small hen's egg, containing a quantity of limpid fluid; and yet these parts had not the least smell of urine. The bladder was entirely sound; there was *nothing* calculous within it, no, not even the stone, of the existence of which Dr. Senter was previously and perfectly satisfied from repeated examinations. At the commencement of her disease, she was five days without passing a drop of urine in any way; this occasioned very great pain and distress, yet the fear of an instrument induced her to withhold a full acknowledgment of her situation from her friends! Ask those to believe this who have suffered the most excruciating tortures from a similar inability. I suspect the girl to have drank her urine. But supposing, merely for the sake of argument, that the urine was actually conveyed to the stomach, either by the blood-vessels and their exhalent terminations, after the regular process of absorption, or by the retrograde action of the absorbents, is it within the bounds of probability, setting impossibility aside, that calculous matter, once deposited in the bladder, should be removed by absorption, whether in a regular or retrograde manner? Would it not rather remain, and become a concrete in its urinary habitation?

Dr. Senter has not mentioned how long he sat to watch the recurrence of the vomiting; and as the disease began in June 1785, and the watching, apparently from the relation of the case, was not commenced until the December following, it is probable that the stomach, from being habituated to the stimulus of the urine, was able to retain it a considerable time,—and thus the vigilance of the doctor might be eluded, by the brackish draught being swallowed before his arrival. This retention of the urine on the stomach would also account for its being occasionally evacuated by the rectum. On the watch, no notice is taken of the state of the bladder after vomiting; and when Dr. Mason saw the vomiting both of urine and gravel, it is not stated that he had been on guard, or what was the previous and subsequent condition of the urinary viscus.

Surely I have now stated enough. But, amid all the wonders of this more than wondrous case, the disappearance of the urinary calculus is not the least remarkable; and Dr. Senter is all amazement in contemplating "by what secret instruments in her extraordinary system" it became decomposed! "'Twas strange, 'twas passing strange!" and I too, like the doctor, should marvel much, did I not feel disposed just to hint that the stone might never have existed. In fine, this case appears to be negligently drawn up, and unaccompanied with that attention to every minute particular particular in all its bearings, which its extraordinary nature ought to have imposed; and the author's remarks upon it prove that medical science, in his hands, must be at least as retrograde as the movements of the absorbent system of Lucy Foster.

The author of the paper on Ischuria, in your Number for May, considers the different ways in which the circulation has got rid of urine, carried into it by the absorbents, as constituting the vis medicatrix naturæ; and he sees no difficulty in understanding how it should be so discharged, any more than any other absorbed fluid which has become useless or noxious to the constitution. This he illustrates by calling to the recollection of his readers instances of dropsies being speedily cured by sudden and spontaneous evacuations of large quantities of fluid from the stomach, skin, and intestines; and concludes that nobody can deny that the fluids which the exhalents had in such instances poured out into the stomach, intestines, and on the skin, were the same which constituted the dropsical swellings thus suddenly disappearing. Whenever the urine finds its way into the blood-vessels, mixing with the vital fluid, and its diffused particles are evacuated at any part with other matters, this should not be stiled the vis medicatrix naturæ; it is a term which ought to be exploded, as it may sometimes lead to the grossest absurdities: for instance, when an aneurism exists, and pointing to the skin, its pressure produces ulceration, and blood is evacuated, the vis medicatrix naturæ, which expression might be employed *as scientifically* on such an occasion, would cut but a sorry figure indeed. A preternatural discharge of urinous particles under ischuria, and an effusion of blood from an aneurism, are consequences impelled by the irresistible laws of animated nature under disease; and one might *as justly* be termed the vis medicatrix naturæ as the other; but this would lead to the odd conclusion—that a preserving and a destroying effect is one and the same. As to large evacuations of fluid from the stomach, or other parts, under dropsical affections, being the identical fluid forming the disease, I think it extremely questionable. They may be explained in another, and, to me, more satisfactory manner. It is no uncommon thing for one diseased action to effect the cure of another; and the existence of a dropsy might be followed by a powerful determination to the stomach, the intestines, or the skin, producing sudden and profuse evacuations; and during such operation, the exhalent and absorbent vessels, connected with the dropsical parts, might be brought into a more healthy condition. Besides, it is well known that both vomiting and purging are powerful ful agents in promoting absorption, and more especially when arising from spontaneity. This appears to me a more rational way of accounting for the casual cure of dropsy by spontaneous evacuation, than by supposing the fluid to be previously absorbed, and then directed to the point of expulsion; and it is more conformable with what is generally observed in the curative process by spontaneous occurrences. A vomiting, a diarrhœa, and an attack of inflammation on the leg, have sometimes proved critical in fever; and the sudden appearance of an eruption on the skin has occasionally removed severe internal disorders, when all the boasted efforts of the healing art have been fruitless and unavailing; but, surely, no medical man will undertake to assert that such incidents were an expulsion of the actual matter of disease.

I am, Sir,
Your obedient Servant,
JOHN PULLEY.

*Bedford,*

